# The Swinging Back of the Pendulum

**Published:** 1922-01

**Authors:** 


### THE SWINGING BACK OF THE PENDULUM

(Oral and Systemic Infections)

Some five years ago attention was directed in these columns to the extremely radical position that was being taken by both the dental and medical professions toward the question of oral focal infection in its relation to systemic disease, and the prophecy was then ventured that in the course of time the pendulum of dental and medical opinion would swing back toward the normal, sane position of equilibrium. In our present issue we take pleasure in presenting a symposium presented before the Dental Society of the State of New York which clearly indicates that the pendulum has surely begun its backward swing to the normal rest position.

The startling revelations resulting from a sober consideration of Hunter's anathematization of crown and bridgework as practiced in Europe by the so-called American dentist stimulated scientific and clinical investigation in both the dental and medical professions. As a result of this investigation the period from 1910 to the present time has been one in which modern, practical procedures of the dental profession have been under severest investigation both from within and without the profession.

Many of these procedures have failed to weather the storm of investigation and have either been modified or discarded and new procedures adopted in conformity to changing opinions during these years, until the present-day procedures and practices in dentistry have been practically revolutionized.

However, the magnified importance attributed to mouth conditions by ill-advised enthusiasts, as the result of this demand, was frequently out of all proportion to the merits of the case; so much so that many dentists and physicians assumed extremely radical positions, dentists advocating and physicians demanding the complete eradication of not only proved focal infectious areas, but all suspected areas on the ground of their possible potentiality for evil; and many such opinions were based upon beneficial results from almost a single case as a criterion.

This last statement is not based on assumption, but upon actual facts. We have had articles presented to this magazine for publication in which the authors have cited two and three, and sometimes single cases in which systemic conditions had been apparently improved by the removal of a pulpless tooth or teeth, the author deducing therefrom the definite conclusion that all pulpless teeth should be removed from the mouth.

Time and experience have enabled the observation of a greater number of cases, and the numerous cases in which the benefit to systemic disease from removal of suspected teeth has been in many instances so convincingly transitory that both dental and medical practitioners have been compelled to halt in their radical advocacy of tooth extraction, and some have faced about even to the extent of recognizing the fact that there are other avenues of infection in the body outside of the oral cavity.

The internist has called attention to the fact that we have in many cases become confused in our conception of the relation of cause and effect, and has pointed out the hitherto apparently overlooked possibility and even actuality of the suspected oral conditions being the result of systemic conditions.

The dental profession for years endeavored to impress upon the medical profession the desirability and necessity of a consideration of the relationship between mouth conditions and systemic conditions, but without any appreciable result until Hunter made his investigations and presented his conclusions; the principal result of the efforts of the dental profession being a tendency to over-exaggerate in their own minds the relationship of oral conditions to systemic disease. When the medical profession's attention was at last directed to this factor in systemic disease and they began to advocate removal of teeth for all conditions not otherwise readily accounted for, the dental profession, or rather its members most actively engaged in promulgating the theory of the relationship between these oral and systemic conditions, in order to be consistent and possibly to more forcibly impress its viewpoint, readily fell into the spirit of the times and even more eagerly than the medical man leaned to wholesale extraction.

The most salutary lesson that the dental profession can learn from this severe schooling, we believe, will be to adopt the method of procedure of the general surgeon, and that is the discriminate selection of cases for operative procedure with a hopeful, not to say assured, chance of success.

In the past the dentist has accepted his mission to be that of saving the teeth of his patients for their masticatory function as a factor and an important one in the process of digestion. We unreservedly believe that to be the mission of dentistry, but imbued with this conception of his mission and with the consideration of his patient's welfare at heart every dentist we feel in the past has endeavored to save hopelessly involved teeth, with the inevitable result of failure and the accompanying cost to the patient.

We do not believe that all teeth can be saved, nor do we believe that all pulpless teeth should be extracted. We believe that many systemic conditions are the result of septic oral conditions, but not all of them. We believe that many pulpless teeth should be removed, but we also believe that many can be and are being saved without in the least endangering the health of the patient. We are conservative just to the extent that conservatism can be intelligently applied to the conservation of salvable teeth, and here is involved the intelligent application of discrimination in the selection of cases that can be operated upon with a reasonable hope of success.

Out of this focal-infection hysteria, from which we have suffered for the past decade, comes at last the mature thought of those who have given much consideration to the question, and we gladly present the conclusions of the four investigators and clinicians above referred to who have labored over the problem in its various aspects. Their conclusions, and we say it in no spirit of boastfulness, fully bear out the contention we have all along made, namely, that teeth can be saved if the available resources of modern dental practice are correctly utilized in the light of our present-day knowledge of oral conditions in their relationship to systemic conditions.

If no other tangible result had come from the confusion caused by focusing the attention of the two professions upon this important problem than that of the realization of the mutual benefits to be derived from a closer affiliation between dentistry and medicine, this alone would seem to justify the trying period of doubt and uncertainty through which only now we are just beginning to see, even though dimly, the dawn of a clearer conception of the inter-relationship between these two branches of the great healing art.—Editorial in *Cosmos,* December, 1921.

